# First records of prevalence and diversity of avian haemosporidia in snipe species (genus *Gallinago*) of Japan

**DOI:** 10.1016/j.ijppaw.2021.07.007

**Published:** 2021-07-20

**Authors:** Mizue Inumaru, Yoshiya Odaya, Yukita Sato, Alfonso Marzal

**Affiliations:** a Laboratory of Biomedical Science, Graduate School of Veterinary Medicine, Nihon University, Fujisawa, 252-0880, Japan; b Abiko City Museum of Birds, 234-3 Konoyama, Abiko, Chiba, 270-1145, Japan; c Department of Zoology, University of Extremadura, 06006, Badajoz, Spain

**Keywords:** Avian haemosporidia, Cytochrome *b*, *Gallinago* snipes, Japan, Migratory birds

## Abstract

Migratory birds are important carriers of pathogens such as viruses, bacteria and protozoa. Avian haemosporidia have been detected from many wild birds of Japan, but the infection status of migratory birds and transmission area are still largely unknown. *Gallinago* snipes are long-distance migratory shorebirds, and five species migrate to or through Japan, including Latham's snipe which is near threatened. Haemosporidian parasites in four snipe species were investigated to understand the role of migratory birds in the transmission of avian haemosporidia. Namely, this study aimed: i) to investigate differences in parasite prevalence and related factors explaining infection likelihood among these migratory species, ii) to explore the diversity in haemosporidian lineages and possible transmission areas, and iii) to assess the possibility of morphological effects of infection. Blood samples were collected from snipes caught in central and southwest Japan during migration. Parasites cyt*b* gene DNA were detected via PCR-based testing, and detected lineages were phylogenetically analyzed. Additionally, factors related to prevalence and morphological effects of infection were statistically tested. 383 birds from four *Gallinago* snipe species were caught, showing higher overall prevalence of avian haemosporidia (17.8 %) than reported in other wader species in previous studies. This high infection rate is presumably due to increased contact with vector insects, resultant of environmental preferences. The prevalence of *Plasmodium* spp. Was higher in Swinhoe's snipes, while *Haemoproteus* spp. Was higher in Latham's snipes. These differences are thought to be related to ecological factors including habitat use, distribution and migratory route. Six lineages detected from juveniles indicate transmission between the breeding and sampling area. Contrary to expectations, a direct link between morphological features and haemosporidian parasite infection were not detected. These findings provide valuable information for conservation of this endangered migratory bird group. Further studies linking biological and parasitological research are anticipated to contribute to conservational actions.

## Highlights

• Prevalence and genetic diversity of avian haemosporidia in *Gallinago* snipes of Japan were investigated for the first time.
• Overall prevalence of avian haemosporidian in snipes (17.8 %) was higher than in other wader species.
• Prevalence and genetic diversity of haemosporidian differed among *Gallinago* species.
• The prevalence of *Plasmodium* was higher in Swinhoe's snipes, while *Haemoproteus* prevalence was higher in Latham's snipes.
• Some haemosporidian lineages suggest host shifts from passeriform birds to the snipes.

## Graphical abstract

**Figure:**
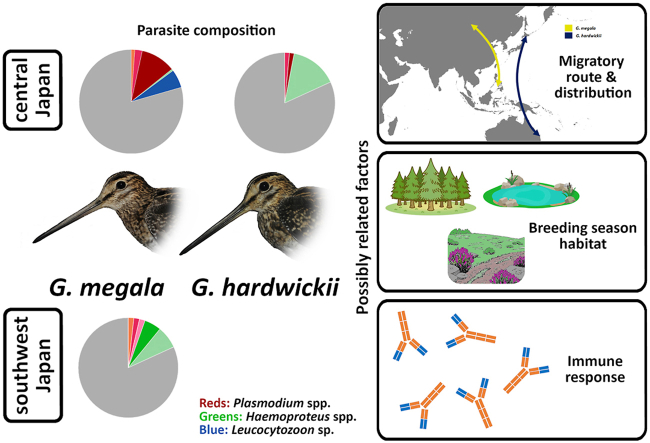

## 1 Introduction

Migratory behaviors are accompanied by the risk of spreading infectious diseases to new areas (Altizer et al., 2011; Rappole et al., 2000; Satterfield et al., 2018). Many studies have suggested that migratory birds have an important role in the transmission of haemosporidian parasites among resident species by carrying new pathogens to a certain area (de Angeli Dutra et al., 2021; Ishtiaq, 2017; Ishtiaq and Renner, 2020; Murata, 2007; Ramey et al., 2015; Waldenström et al., 2002). Meanwhile, some studies suggest a reduced introduction of new pathogens due to migratory species because of limitations in local host assemblage of co-transported parasites (e.g. the presence of viable vector species, host specificity and migratory timing) (Hellgren et al., 2013; 2007; Pulgarín-R et al., 2019; Ricklefs et al., 2017; Soares et al., 2020). Nonetheless, migratory behaviors are closely linked to parasitism, as different migratory populations within a species may encounter different parasites through different migratory routes (Cumming et al., 2013; Ramey et al., 2015; Shurulinkov et al., 2012). There is also a trade-off between the risk of infection and risk of migration which may ultimately lead to modifications and adaptations of migratory routes (Clark et al., 2016; Mendes et al., 2005; Sorensen et al., 2019; Waldenström et al., 2002). Such global patterns in parasite distribution can be used to reveal possible locations of transmission (Inumaru et al., 2017; Ishtiaq, 2017; Ishtiaq et al., 2007; Valkiūnas, 2005; Waldenström et al., 2002).

Avian haemosporidia have been detected from various wild birds of Japan (Imura et al., 2012; Inumaru et al., 2017; Murata, 2002, 2007; Murata et al., 2007; Sato et al., 2007; Tanigawa et al., 2013; Yoshimura et al., 2014). However, information on the infection status in migratory birds of Japan remains limited (Inumaru et al., 2017; Murata, 2002, 2007; Tanigawa et al., 2013; Yoshimura et al., 2014). This is especially relevant in the case of species belonging to the genus *Gallinago*, where most species have not been previously investigated for malaria infection. Although *Gallinago* genus includes 17 species distributed across many continents, the prevalence and genetic diversity of haemosporidian parasites have been reported in only two *Gallinago* species (*G. gallinago* and *G. media*) (Halvarsson, 2016; Höglund et al., 2017; Pardal et al., 2014) (MalAvi database Version 2.4.8 Feb 25th, 2021 (Bensch et al., 2009),).

*Gallinago* species, commonly referred to as snipes, are distributed throughout the world (Gill et al., 2020), including five species that migrate to or through Japan. The common snipe (*G. gallinago*) and solitary snipe (*G. solitaria*) are known to winter in parts of Japan. Latham's snipe (*G. hardwickii*) breeds mainly in northern parts of Japan and winters in parts of Australia. Meanwhile, the Swinhoe's snipe (*G. megala*) and pin-tailed snipe (*G. stenura*) are passage migrants, breeding in areas north of Japan and wintering in areas such as South East Asia and Australia (Brazil, 2009; Hayman et al., 1986; Message and Taylor, 2005). Japan therefore has a different but equally important role for these snipe species. Due to habitat loss and hunting, the common snipe and Latham's snipe are decreasing in population (IUCN, 2020; Kitajima and Fujimaki, 2003; Ura, 2007). Particularly, Latham's snipe is listed as near threatened in Japan and parts of Australia (Department of the Environment, 2020; Ministry of the Environment, 2019); and conservation projects have been dedicated to support this species (CeRDI, 2020; Wild Bird Society of Japan, 2020). Swinhoe's snipe and pin-tailed snipe have been given much less attention and population trends are unknown.

While haemosporidian infection can be subclinical, many studies have revealed the risk of infection, with the most extreme risk being death. Species that have no evolutionary history or only a short history in the presence of vectors such as mosquitoes have little to no tolerance of infectious diseases transmitted by these vectors. This is the case with the native honeycreepers of Hawai'i which were naïve to introduced avian malaria, leading to population decline and even the extinction of many native bird species (Atkinson et al., 2013; Atkinson and Lapointe, 2009; LaPointe et al., 2012; Van Riper et al., 1986). Captive birds such as those in zoos and aviaries are also at high risk, as they may encounter haemosporidian parasites that they would not have encountered in their original distribution (Inumaru et al., 2021; Lee et al., 2018; Olias et al., 2011; Scott, 1927; Vanstreels et al., 2016). Apart from those lethal effects, some studies have also reported negative associations between haemosporidian infection and host status, including both morphological and physiological effects. These effects include decreased body mass (Coon et al., 2016; Fleskes et al., 2017; Marzal et al., 2008), delayed molt (Morales et al., 2007), shorter wing length (Dunn et al., 2013), slower feather growth rate (Coon et al., 2016; Marzal et al., 2013), impaired reproductive success (Höglund et al., 2017; Merino et al., 2000) and reduced fitness (Merino et al., 2000; Palinauskas et al., 2008). In some *Gallinago* species, male snipes compete for mating partners through display flights and intense fights (Byrkjedal, 1990; Golovina, 1998; Hayman et al., 1986; Morozov, 2004; Nakamura and Shigemori, 1990). In these energy-demanding behaviors, maintaining good physical conditions is crucial to win partners. Additionally, during display flights, snipes use their tail feathers to create characteristic buzzing sounds (Byrkjedal, 1990; Morozov, 2004; Nakamura and Shigemori, 1990), and it is suggested that the tail length and number of feathers may have impacts on mating success (Ura et al., 2005). If such negative effects are present in infected snipes, they may indirectly affect reproductive success.

In this study, avian haemosporidian parasites in four species of snipes were investigated in order to gain basic knowledge of infection in these species, which may indirectly contribute to the conservation of these declining species. Namely, this study aimed: i) to investigate the parasite prevalence and factors affecting the prevalence among these migratory species, ii) to explore the diversity in haemosporidian lineages and possible areas of transmission, and iii) to assess the possibility of morphological and physiological effects of infection, particularly those related to reproductive behaviors.

## 2 Materials and methods

### 2.1 Sample collection

**Fig. 1:**
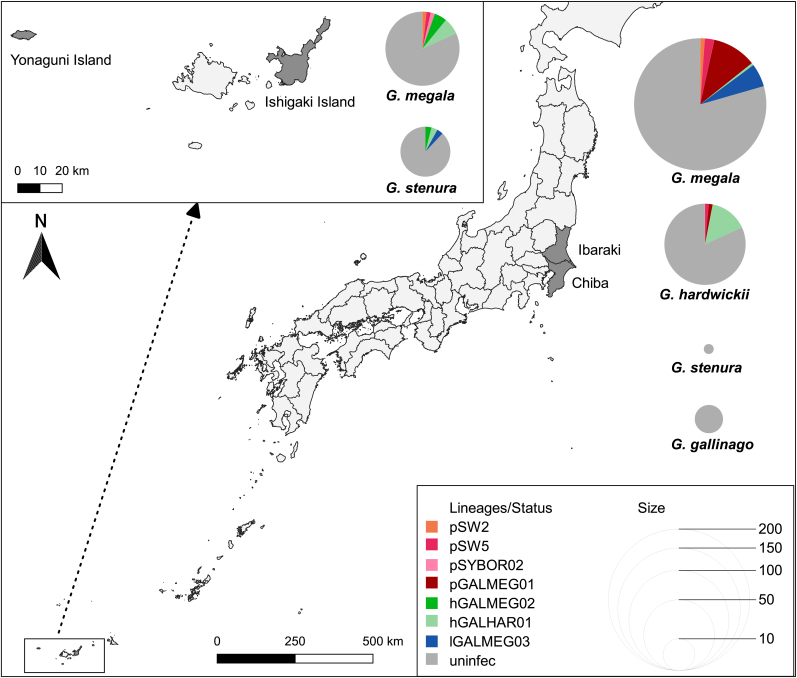
Map of sampling areas, including the prevalence and lineage composition of each area by host species.

Swinhoe's snipes, Latham's snipes, pin-tailed snipes and common snipes were captured from 2012 to 2020 at two distinct areas of central and southwest Japan nearly 2000 km apart (Fig. 1). In central Japan, we collected snipes at multiple localities of Chiba (35°36′N 140°07′E) and Ibaraki (36°33′N 139°53′E) prefectures, mostly during the spring and autumn migration from April to May and August to October, respectively. In southwest Japan, birds were caught in the autumn at two islands, Ishigaki Island (24°20′N 124°09′E) and Yonaguni Island (24°27′N 122°55′E). The birds were caught at night either using a scoop net and flashlight or by mist nets. Species and age of the collected birds were determined according to plumage criteria (Hayman et al., 1986). The captured snipes were fitted with a metal ring with distinct identification numbers. The following measurements were collected for each individual: maximum wing length (to the nearest 1 mm), tail length (to the nearest 1 mm), outermost tail length (to the nearest 1 mm), tarsus length (to the nearest 0.1 mm), exposed culmen (to the nearest 0.1 mm), fat score (on a scale of 1–5), body mass (to the nearest 1 g) and molt score (on a scale of 0–50) (Ginn and Melville, 1983). After data collection, a small amount of blood was taken from the brachial vein. The blood was placed in microtubes containing 70–99.5 % ethanol and sent to Nihon University, College of Bioresource Sciences, Department of Veterinary Medicine, Laboratory of Biomedical Science, then kept at −20 °C until further processes. Blood smears were not prepared in this study. The birds were released after data collection and blood sampling.

All procedures for collecting samples from birds in this study were performed in accordance with the ethical standards of the Act on Welfare and Management of Animals (1973). No birds were harmed during sampling.

### 2.2 DNA extraction and molecular sexing

DNA was extracted from the blood samples using standard phenol-chloroform method, with tris-EDTA as the final buffer. DNA concentration was confirmed using Nanodrop One Microvolume UV–Vis Spectrophotometers (Thermo Fisher Scientific, MA, USA) and adjusted to a final concentration of 50 ng/μl. For Swinhoe's snipes and Latham's snipes, molecular sexing was done using a previously-described polymerase chain reaction (PCR) protocol targeting the chromo-helicase DNA (*CHD*) *1* gene (Fridolfsson and Ellegren, 1999; Ura et al., 2005). Using the primers 2550 F and 2718 R, a PCR reaction was carried out. The reaction mixture included 2 mM MgCl_2_, 0.2 mM deoxynucleotide triphosphate, 10xExTaq buffer (Mg^2+^ free; Takara, Ohtsu, Japan), 0.625U Ex-Taq (Takara), 0.6 μM each primer and 50 ng of template DNA, making the final volume 25 μl each. PCR cycle conditions were according to the original manuscript (Fridolfsson and Ellegren, 1999). Because DNA from anatomically sexed snipes were not available, DNA from one male and one female herring gull (*Larus argentatus*) of a previous study (Inumaru et al., 2017) were included as positive controls. Negative controls using distilled water instead of DNA were also included. Visualization of the PCR products were done using 1.5 % agarose gels (Agarose S: Nippon Gene, Tokyo, Japan) containing ethidium bromide (Nacalai tesque, Kyoto, Japan). Electrophoresis was done in chambers containing TAE buffer at 100 V for about 20 min. Gels were visualized under ultraviolet light.

### 2.3 Molecular screening of avian haemosporidia

A nested-PCR targeting the partial mitochondrial cytochrome *b* (cyt*b*) gene of avian haemosporidia was carried out using a previously described protocol (Hellgren et al., 2004). The composition of the reaction mixture was the same as the molecular sexing described above (see section 2.2). As a positive control, *Plasmodium gallinaceum* GALLUS01 derived from an experimentally infected chicken (*Gallus gallu*s) and *Leucocytozoon* sp. OTULEM04 from a Sunda scops-owl (*Otus lempiji*) rescued in central Japan were included. A negative control containing distilled water instead of DNA was also prepared. One positive and one negative control were included in each gel. Visualization was done in the same method as molecular sexing. No negative controls showed any amplification. Positive samples were cut out of the gel and DNA was extracted using Thermostable β-Agarase (Nippon Gene, Chiyoda, Japan).

### 2.4 Phylogenetic analysis of the detected haemosporidia

Extracted DNA was directly sequenced in both directions with BigDye™ terminator cycle sequence kit (Ver 3.1 Applied Biosystems, Förster City, CA, USA) and an ABI 3130-Avant Auto Sequencer (Applied Biosystems). The obtained sequences were assembled and compared with other sequences in the GenBank database using the Basic Local Alignment Search Tool (Madden, 2013) and sequences in the MalAvi database (Bensch et al., 2009). All samples that had low-quality reads or were not 100 % identical to a previously identified lineage were re-tested by PCR in order to remove any possible false positives. Detected lineages that were not identical to previously identified lineages were translated into amino acid sequences using MEGA X to check for possible sequence errors (Kumar et al., 2018).

For phylogenetic analysis, morphologically identified lineages of the three haemosporidian genera and molecularly close lineages were aligned with the detected lineages to construct a Bayesian phylogeny. Pairwise distance between lineages was calculated using the Kimura-2-parameter model of substitution in MEGA X (Kumar et al., 2018). *Theileria annulata* was included as an outgroup. Model selection was done using ModelFinder in IQ-TREE 1.6.12 (Kalyaanamoorthy et al., 2017). A bayesian phylogeny was constructed with Mr. Bayes version 3.2 (Ronquist et al., 2012) using the General Time Reversible model with gamma distribution for variable sites and proportion of sites as invariable (GTR+Γ+I), as implemented by ModelFinder under Bayesian Information Criterion (Kalyaanamoorthy et al., 2017). Two independent runs of Markov Chain Monte Carlo (MCMC) sampling were done for three million generations, sampling every 1000 generations (Ronquist et al., 2012). As a burn-in step, the first 25 % of the trees were discarded. The final tree was visualized with FigTree 1.4 (Rambaut, 2012).

### 2.5 Statistical analysis

Prevalence of avian haemosporidia was compared between species with Fisher's exact test. Following, a post-hoc multiple comparison test with Bonferroni correction was carried out. We then used general linear models (GLM) with binomial distribution and logit function to test whether species, sex, age, sampling area, and season had impacts on infection status. For all GLM tests, each parasite genus was tested individually. For age, the birds were classified in to either juveniles (juvenile to first winter plumage) or adults (first summer to adult plumage). No interaction effects were significant and were removed from the models. Pin-tailed snipes and common snipes were removed from all tests due to the small sample size, and GLM tests were carried out between Swinhoe's and Latham's snipes.

To test for differences in morphological traits, Welch's t-tests were performed between infected and uninfected individuals. Tail length, length of outermost rectrix, tarsus length, fat score and body mass were tested. Only adults of Swinhoe's snipe and Latham's snipe caught in central Japan were used for analysis. Juveniles were excluded because adults and juveniles are known to have varying biometrics (Prater et al., 2007). Because sexual dimorphism is known in these species (Frith et al., 1977; Prater et al., 2007; Ura et al., 2005), males and females were individually tested. Additionally, because seasonal variations in metabolic rates are known in many species (Bairlein, 2002; Frith et al., 1977; Jenni-Eiermann et al., 2002; Kvist and Lindström, 2001), fat score and body mass were evaluated separately for autumn and spring. For groups that had only one or less infected individual, comparisons were not possible. All statistical analyses were conducted in the software R ver. 3.6.3 (R Core Team, 2020). The package ‘fmsb’ was used for the post-hoc multiple comparison test (Nakazawa, 2019). Statistical values are rounded to the third decimal and the 5 % significance level was used.

## 3 Results

**Fig. 2:**
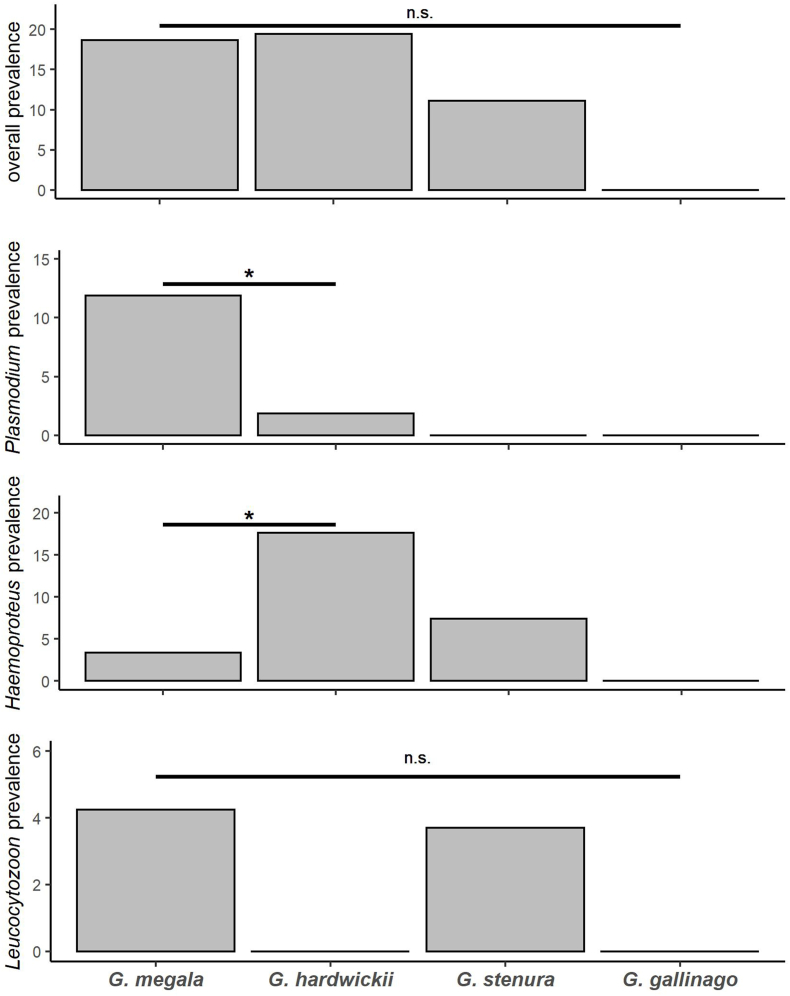
Haemosporidian parasite prevalence among snipe species. Asterisk (*) indicates significant differences (p < 0.05), and n. s. indicates no significant differences (p ≥ 0.05).

In total, 383 birds were caught at the two areas (Table 1). All four species were caught in central Japan, while only two species were caught in southwest Japan. 68 birds were positive by PCR for any haemosporidia (overall prevalence = 17.8 %) (Table 1). All common snipes were negative for haemosporidia by PCR. There was no difference in overall prevalence among Swinhoe's (18.6 %), Latham's (19.4 %) and pin-tailed snipes (11.1 %) (Fisher's exact test: *p* = 0.327, Fig. 2). However, when comparing each genus separately, there was a significant difference among species for *Plasmodium* and *Haemoproteus* (Fisher's exact test: *Plasmodium p* = 0.003, *Haemoproteus p* < 0.001). Specifically, Plasmodium prevalence was higher for *G. megala* than for *G. hardwickii*, whereas *Haemoproteus* prevalence was significantly higher for *G. hardwirckii* than for *G. megala* (Multiple comparison with Bonferroni correction: *Plasmodium* p = 0.009, *Haemoproteus* p < 0.001; Fig. 2). There was no significant difference among species for *Leucocytozoon* prevalence (Fisher's exact test: p = 0.110; Fig. 2).

**Table 1:** PCR results of haemosporidian detection per sampling location and season in snipes from this study.

		autumn						spring						Total					
area	Species	number sampled	PCR positive (%)	P^a^	H^a^	L^a^	P/L^a^	number sampled	PCR positive (%)	P^a^	H^a^	L^a^	P/L^a^	number sampled	PCR positive (%)	P^a^	H^a^	L^a^	P/L^a^
central Japan	*Gallinago megala*	175	34 (19.4)	25	1	10	2	6	0	0	0	0	0	181	34 (18.8)	25	1	10	2
	*Gallinago hardwickii*	66	12 (18.2)	2	10	0	0	42	9 (21.4)	0	9	0	0	108	21 (19.4)	2	19	0	0
	*Gallinago stenura*	1	0	0	0	0	0	1	0	0	0	0	0	2	0	0	0	0	0
	*Gallinago gallinago*	8^b^	0	0	0	0	0	4	0	0	0	0	0	12	0	0	0	0	0
	sub-total	250	46 (18.4)	27	11	10	2	53	9 (17.0)	0	9	0	0	303	55 (18.2)	27	20	10	2
southwest Japan	*Gallinago megala*	55	10 (18.2)	3	7	0	0	0	–	–	–	–	–	55	10 (18.2)	3	7	0	0
	*Gallinago stenura*	25	3 (12.0)	0	2	1	0	0	–	–	–	–	–	25	3 (12.0)	0	2	1	0
	sub-total	80	13 (16.3)	3	9	1	0	0	–	–	–	–	–	80	13 (16.3)	3	9	1	0
	Total	330	59 (17.9)	30	20	11	2	53	9 (17.0)	0	9	0	0	383	68 (17.8)	30	29	11	2
																			

a P:Plasmodium sp.; H:Haemoproteus sp.; Leucocytozoon sp.; P/L: co-infection between Plasmodium sp. and Leucocytozoon sp.

b Two individuals were captured in the winter (November and February).

Species was a significant factor in all tested GLM models of *Plasmodium* and *Haemoproteus* (Table 2). Accurate results could not be obtained for *Leucocytozoon* due to the high standard error. Sex, age, and season did not explain the likelihood of becoming infected by any haemosporidian genera. However, note the high standard error in the season model for *Plasmodium* spp. The prevalence among Swinhoe's snipes captured in autumn significantly differed between sampling areas for *Haemoproteus* spp., being higher for snipes captured in southwest Japan (12.73 %) than from individuals from central Japan (0.57 %) (Table 1, Table 2).

**Table 2:** Models and their coefficients for the General Linear Models (GLMs) to test host factors associated with haemosporidian prevalence in snipes.

		Plasmodium				Haemoproteus				Leucocytozoon			
Model	Coefficients	Estimate	SE	z value	Pr (>|z|)	Estimate	SE	z value	Pr (>|z|)	Estimate	SE	z value	Pr (>|z|)
Species	(intercept)	−3.970	0.714	−5.563	<0.01	−1.544	0.253	−6.110	<0.01	−20.570	1706.110	−0.012	0.990
	*G. megala*	1.965	0.742	2.650	**0.008**	−1.806	0.440	−4.108	**<0.01**	17.450	1706.110	0.010	0.992
species × sex^a^	(intercept)	−3.841	0.744	−5.165	<0.01	−1.483	0.346	−4.281	<0.01	−3.045	0.418	−7.286	<0.01
	*G. megala*	1.933	0.743	2.600	**0.009**	−1.821	0.444	−4.101	**<0.01**				
	male	−0.232	0.396	−0.587	0.557	−0.107	0.418	−0.255	0.799	−0.174	0.659	−0.264	0.791
species × age^a^	(intercept)	−4.276	0.779	−5.489	<0.01	−1.335	0.347	−3.851	<0.01	−3.701	0.716	−5.171	<0.01
	*G. megala*	1.957	0.742	2.638	**0.008**	−1.800	0.440	−4.090	**<0.01**				
	adult	0.456	0.434	1.049	0.294	0.352	0.418	−0.842	0.400	0.804	0.803	1.002	0.316
area^b^	(intercept)	−1.792	0.216	−8.294	<0.01	−5.159	1.003	−5.144	<0.01	−2.803	0.326	−8.608	<0.01
	southwest Japan	−1.061	0.632	−1.679	0.093	3.234	1.081	2.990	**0.003**	−16.763	1450.071	−0.012	0.991
season^c^	(intercept)	−3.466	0.718	−4.826	<0.01	−1.723	0.344	−5.018	<0.01				
	spring	−17.100	2735.856	−0.006	0.995	0.424	0.510	0.832	0.406				

a Leucocytozoon was tested only among G. megala.

b Only G. megala caught in autumn were included.

c Only G. hardwickii caught in central Japan were included. Leucocytozoon was not detected and was not tested.

**Fig. 3:**
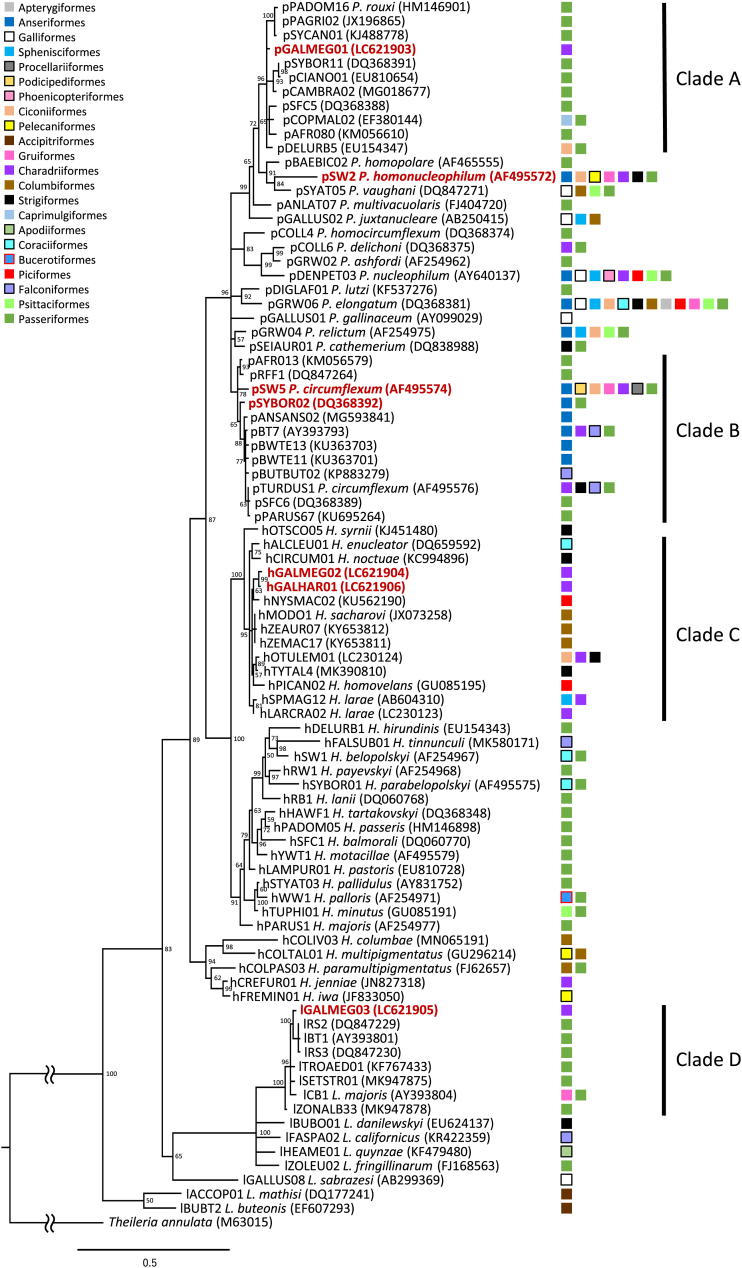
Bayesian phylogenetic analysis of cyt*b* gene lineages (470 bp) of avian haemosporidian parasites, rooted with *Theileria annulata*. Posterior clade probabilities of >0.60 were indicated. The branch lengths are drawn proportionally to the amount of change according to the substitution model applied. Lineages derived in this study are shown in red letters. Major clades (A–C) containing derived lineages are shown. The host order is shown to the right of the lineage name, according to the provided legend. (For interpretation of the references to colour in this figure legend, the reader is referred to the Web version of this article.)

Detected parasites were identified as seven lineages consisting of four *Plasmodium* spp., two *Haemoproteus* spp. and one *Leucocytozoon* spp. lineage(s) (Fig. 3, Table 3). Of these, three *Plasmodium* spp. lineages were previously known lineages, while the other four were detected for the first time. These new lineages were named according to MalAvi database (Bensch et al., 2009) and deposited in GenBank database (NCBI website, http://www.ncbi.nlm.nih.gov/BLAST) under accession numbers **LC621903**-**LC621906** (Table 3). All seven lineages were detected from Swinhoe's snipe while three each were detected from Latham's and pin-tailed snipe. Five lineages were detected from central Japan and six lineages were detected from southwest Japan, including four that were detected in both areas (Fig. 1, Table 3). Also, six lineages were detected in juveniles, including *P. homonucleophilum* pSW2 and *Plasmodium* sp. SYBOR02, which were solely detected from juveniles. The clades A and D contain lineages that have previously been detected predominantly by passeriform birds (Fig. 3). Meanwhile, clade B and C included lineages from various host orders, including Charadriiformes. By pairwise distance, species closest to the newly detected lineages of *Plasmodium*, *Haemoproteus* and *Leucocytozoon* were *P. rouxi* (pPADOM16, HM146901), *H. larae* (hSPMAG12, AB604510) and *L. majoris* (lCB1, AY393804), respectively.

**Table 3:** Parasite lineages, genus, GenBank accession numbers and number of infected adult and juvenile snipes detected per sampling location and bird species in this study.

genus	lineage	accession no.	*Gallinago megala*				*Gallinago hardwickii*		*Gallinago stenura*
			central Japan		southwest Japan		central Japan		southwest Japan
			adult	juvenile	adult	juvenile	adult	juvenile	juvenile
*Plasmodium*	pSW2	AF495572		2		1			
	pSW5	AF495574	1	3	1			1	
	pSYBOR02	DQ368392				1			
	pGALMEG01^a^	LC621903	19^b^				1		
*Haemoproteus*	hGALMEG02^a^	LC621904			2	1			1
	hGALHAR01^a^	LC621906	1		1	3	11^c^	8	1
*Leucocytozoon*	lGALMEG03^a^	LC621905	8^b^	2					1

a Novel lineages.

b 2 individuals were infected by both pGALMEG01 and lGALMEG03.

c 9 individuals were caught in the spring. All others were caught in the autumn.

No significant difference between infected and uninfected individuals was seen among all tested morphological traits (Table 4).

**Table 4:** Mean values of morphological measurements in infected and uninfected snipes, and *t*-test results.

				mean		*t*	df	*p*
				infected	uninfected			
Tail length								
	*G. megala*							
		male		60.182 (±1.662)	60.345 (±2.303)	−0.278	18.642	0.784
		female		57.313 (±1.448)	58.088 (±1.640)	−1.837	26.816	0.077
	*G. hardwickii*							
		male		67.571 (±2.370)	66.968 (±1.722)	0.637	7.494	0.543
		female		65.400 (±2.074)	64.905 (±1.786)	0.492	5.504	0.642
Length of outermost rectrix								
	*G. megala*							
		male		55.636 (±1.963)	56.182 (±2.302)	−0.816	16.031	0.426
		female		51.688 (±2.469)	51.368 (±1.809)	0.482	19.741	0.635
	*G. hardwickii*							
		male		63.143 (±3.024)	62.839 (±3.760)	0.229	10.662	0.823
		female		57.250 (±2.754)	57.762 (±3.223)	−0.331	4.722	0.755
Tarsus length								
	*G. megala*							
		male		34.836 (±1.049)	34.907 (±1.234)	−0.198	16.073	0.845
		female		36.550 (±1.176)	36.472 (±1.191)	0.234	24.337	0.817
	*G. hardwickii*							
		male		36.557 (±1.474)	35.775 (±0.988)	1.340	7.224	0.221
		female		36.120 (±1.262)	36.943 (±1.153)	−1.332	5.704	0.234
Fat score								
	*G. megala*							
		male	autumn	3.182 (±0.751)	3.000 (±1.066)	0.672	19.656	0.509
		female	autumn	3.500 (±1.095)	3.278 (±0.920)	0.738	21.653	0.468
	*G. hardwickii*							
		male	autumn	3.500 (±0.707)	3.583 (±1.165)	−0.138	2.070	0.902
			spring	1.600 (±0.548)	1.300 (±0.470)	1.126	5.570	0.307
		female	spring	1.250 (±0.500)	1.615 (±0.650)	−1.185	6.497	0.278
Body mass								
	*G. megala*							
		male	autumn	166.364 (±24.703)	171.269 (±27.200)	−0.588	15.586	0.565
		female	autumn	189.250 (±28.193)	191.185 (±30.961)	−0.236	26.671	0.816
	*G. hardwickii*							
		male	autumn	204.500 (±21.920)	208.000 (±30.591)	−0.196	1.738	0.865
			spring	142.000 (±21.714)	126.300 (±12.079)	1.558	4.637	0.185
		female	spring	135.250 (±2.363)	138.583 (±20.007)	−0.565	11.864	0.582

## 4 Discussion

### 4.1 Comparison of parasite prevalence among waders

Although *Plasmodium* sp. has previously been reported from two common snipes and one Latham's snipe (Murata, 2002, 2007), this study reports the prevalence and genetic diversity of *Gallinago* snipes in Japan for the first time. Overall, avian haemosporidia was detected from 17.8 % of the individuals (Table 1). This prevalence is similar to the prevalence of 16.5–30 % reported in great snipes (Halvarsson, 2016; Höglund et al., 2017). However, other studies reported lower parasite prevalence in other wader species (Clark et al., 2016; Martínez-De La Puente et al., 2017; Mendes et al., 2005; Pardal et al., 2014). For example, a comparative analysis across 46 species of five continents using a global database revealed an average prevalence of 6.2 % for wader species (Clark et al., 2016). The lower prevalence found in these birds has been explained by ecological factors such as their habitat use and migration strategies (Clark et al., 2016; Mendes et al., 2005). Following this idea, marine species are generally known to have an extremely low parasite prevalence because the saline environment is not suitable for vector insects of avian haemosporidia (Clark et al., 2016; Martínez-De La Puente et al., 2017; Mendes et al., 2005). On the contrary, snipe species (such as those from our study) inhabit open woodlands near streams and freshwater wetlands such as rice paddies and meadows (Brazil, 2009; Hayman et al., 1986; Message and Taylor, 2005; Ura, 2007), which are suitable environments for haemosporidian vectors such as mosquitoes (Dale and Knight, 2008; Ferraguti et al., 2016; Gimonneau et al., 2012; Richards et al., 2010). Hence, the high prevalence in snipes compared to other wader species may be due to increased contact with vector insects. However, other explanations including taxonomical differences and immunocompetence have also been suggested (Martínez-Abraín et al., 2004).

### 4.2 Comparison of parasite prevalence within snipe species

We found no significant difference in overall prevalence between Swinhoe's and Latham's snipes. However, the prevalence of *Plasmodium* spp. Was higher in Swinhoe's snipes, while *Haemoproteus* spp. Prevalence was higher in Latham's snipes (Fig. 2, Table 2). Similarly, differences in parasite genus composition have been recorded in closely related host species (Dubiec et al., 2016; Scordato and Kardish, 2014; Smith et al., 2018). We propose some non-mutually exclusive alternatives to explain these differences in parasite prevalence.

First, host ecology including migratory distributions, timing of migration, and habitat preferences may be associated to parasite prevalence, in relation to contact with vectors. For example, the migratory distributions differ among snipe species. Latham's snipes breed primarily in northernmost Japan and parts of Russia, as well as in selective highlands in areas further south (Frith et al., 1977; Hayman et al., 1986; Ura, 2007), and migrate south to their wintering sites in eastern Australia (CeRDI, 2020; Frith et al., 1977; Ura, 2007). Meanwhile, Swinhoe's snipes breed throughout a wide range in parts of Russia and Mongolia, and migrate south through eastern Mongolia, China, and Japan. The main wintering range lies in Southeast Asia (Leader and Carey, 2003; Morozov, 2004), although small populations have also been periodically recorded in northern Australia and other parts of Melanesia (Frith et al., 1977; Hayman et al., 1986). Differences in parasite prevalence have been detected in populations or species that have different migratory routes (Pedro et al., 2019; Valkiūnas and Iezhova, 2001). Additionally, the timing of migration also differs between snipe species. Latham's snipes begin leaving their breeding grounds from mid-July to August, earlier than Swinhoe's snipes which begin leaving from early August to September (Frith et al., 1977; Golovina, 1998; Hayman et al., 1986; Leader and Carey, 2003). Furthermore, these two species use similar environments during migration when rice paddies and freshwater wetlands are favorable (Brazil, 2009; Hayman et al., 1986; Message and Taylor, 2005; Ura, 2007). Meanwhile, habitat usage differs during the breeding season, as Swinhoe's snipes prefer a wide variety of habitats from open woodlands near river valleys and marshes to taiga and forest-steppe zones, while Latham's snipes prefer drier grasslands and heathlands from low to high elevations (Brazil, 2009; Frith et al., 1977; Golovina, 1998; Hayman et al., 1986; Leader and Carey, 2003; Message and Taylor, 2005). Selective feeding of certain host species in response to host preferences and availability have also been reported in vector species (Kim and Tsuda, 2010; Medeiros et al., 2015; Santiago-Alarcon et al., 2013). Each of these ecological factors are strongly correlated with how likely the birds are to come in contact with vectors (Elbers et al., 2015; Richards et al., 2010; Satterfield et al., 2018), and can therefore influence contact with haemosporidian parasites (Ágh et al., 2019; Chahad-Ehlers et al., 2018; Kim and Tsuda, 2010; Lalubin et al., 2013; Sol et al., 2000). However, vector and parasite abundance of the areas inhabited by each snipe species are not known. Moreover, the area of transmission would be crucial to further investigate these differences.

Alternatively, the difference in prevalence among species may be explained by differences in immune response. Immune response may function in a number of different ways. Hosts may be capable of tolerating infection, keeping fitness costs at minimum. Another strategy would be for the host to be able to reduce or even clear the infection (Delhaye et al., 2018; Krams et al., 2012; Møller and Erritzée, 1998; Sorci, 2013; Sorensen et al., 2016). Differences in immune response may be resultant of life-history traits such as habitat selection pressure and evolutionary history, which are linked to parasite exposure (Atkinson et al., 2013; Boyd et al., 2018; Grilo et al., 2016; Lee, 2006; Mendes et al., 2006). However, the response is not necessarily consistent, as some species, or even individuals within a species, may cope with the infection by keeping a steady infection level while others may completely clear the infection from their bodies (Atkinson et al., 2013; Lee, 2006; Møller and Erritzée, 1998). The prevalence in Swinhoe's snipe and Latham's snipe may therefore differ not only by ecological factors such as habitat and distribution, but also by physiological aspects such as immunological factors (Martínez-Abraín et al., 2004). However, this possibility should be considered with caution, as differences in immune response between *Plasmodium* and *Haemoproteus* parasites have not been well documented. Furthermore, possible co-infections of the two parasite genera are difficult to detect by molecular methods (Bernotienė et al., 2016; Valkiūnas et al., 2006), and blood smears were not investigated in this study. These limitations should also be considered.

It was not possible to statistically analyze pin-tailed snipes and common snipes in this study, due to considerably small sample size compared to the other two species. Further sampling of these two species would be needed to reveal a more accurate population prevalence of avian haemosporidia.

### 4.3 Other factors in relation to parasite prevalence

Sex and age did not explain variation in the probability of infection of each parasite genus. These findings are similar to many previous studies reporting that sex is not a significant factor to explain blood parasite infection (Ágh et al., 2019; Granthon and Williams, 2017; Halvarsson, 2016; Pedro et al., 2019; Podmokła et al., 2014), but see (Baillie et al., 2012; Jones, 2019). There were no age-related differences observed in this study, in accordance with some previous studies (Granthon and Williams, 2017; Pedro et al., 2019). However, other studies have revealed higher prevalence in adults, probably due to accumulation of infection in adults and higher mortality in younger birds (Mendes et al., 2005; Podmokła et al., 2014; Sol et al, 2000, 2003). Interestingly, the parasite prevalence for great snipes was lower in adults compared to juveniles (Halvarsson, 2016), suggesting differences in immune systems and protective behaviors (Mendes et al., 2005). As no difference between age was observed in this study, juveniles and adults of these snipe species may be equally exposed to vectors. This is also suggestive that juveniles may equally contribute to the dispersal and transmission of parasites between regions on their first migratory flight (Pulgarín-R et al., 2019).

For Swinhoe's snipes, *Haemoproteus* prevalence varied between populations, being higher for snipes captured in southwest Japan compared to individuals from central Japan. Additionally, although not statistically significant, Swinhoe's snipes caught in central Japan had a higher *Plasmodium* prevalence compared to those of southwest Japan. In a previous study, morphological differences were observed between Swinhoe's snipes captured in these two areas, which suggests that these are different populations (Odaya et al. unpublished). These populations may possibly have different migratory pattern, and hence these disparities in parasite prevalence can be attributed to differences in vector and/or parasite exposition between populations, as discussed above (see section 4.2).

In Latham's snipes, there was no seasonal effect on *Haemoproteus* spp. Prevalence. These findings are consistent with previous studies, which have found similar parasite prevalence during the fall and spring of long-distance migrants (Hellgren et al., 2013; Pulgarín-R et al., 2019).

### 4.4 Parasite lineage composition

In general, haemosporidian parasites infect closely related host species, with frequent host switches among those related species (Clark and Clegg, 2017; Ellis and Bensch, 2018; Hellgren et al., 2007; Pulgarín-R et al., 2018; Ricklefs et al., 2014; Ricklefs and Fallon, 2002; Santiago-Alarcon et al., 2014). Nevertheless, host shifts of generalist parasites among more distant species, including species of different orders, have also been reported (Clark and Clegg, 2017; Ricklefs et al., 2014; Santiago-Alarcon et al., 2014). Of the seven identified lineages, four lineages were detected for the first time. Interestingly, pGALMEG01 and lGALMEG03 were placed in clades with lineages detected predominantly from passeriform birds (Fig. 3). Multiple individuals were infected with each of these two lineages, considerably decreasing the possibility of an accidental spillover from passeriform birds. Rather, it seems more likely that these lineages have undergone host shifts from passeriform birds to these snipes. However, sampling bias must also be considered, as passeriform birds are relatively easier to sample and have thus been more investigated compared to birds of other orders (Clark et al., 2014). Meanwhile, the *Plasmodium* clade B and *Haemoproteus* clade C consisted of host birds belonging to various orders including charadriiformes. Although the lineage pSYBOR02 has been predominantly detected from passeriform birds, close lineages including SW5 of *P. circumflexum* have been detected from various host species. By pairwise distance, the closest morphological species to the detected *Haemoproteus* lineages was *H. larae*. This species has been detected by microscopy in various charadriiform birds (Peirce, 1981; Valkiūnas, 2005; Yakunin, 1972) and was recently genetically described from rescued charadriiform birds (Inumaru et al, 2017, 2020). In this study, blood slides were not obtained, and morphological identifications could not be made. However, from the combination of host and molecular information, there is a possibility that these detected lineages may be another molecular variant of *H. larae*. Meanwhile, *H. scolopaci*, *H. contortus*, and *H. rotator* are species that have previously been morphologically detected in *Gallinago* snipes of the Philippines, including Swinhoe's snipes and pin-tailed snipes (Valkiūnas, 2005). These three parasite species have not been molecularly described yet, and thus lineages in this study cannot be compared. Future studies analyzing blood smears would provide new insights.

We also found differences in the number of detected haemosporidian lineages among snipe species. Swinhoe's snipes exhibited the highest parasite diversity (seven lineages), whereas only three lineages each were detected from Latham's snipes and pin-tailed snipes (Table 3). While several known parasite species such as *Plasmodium relictum* and *P. elongatum* are widely distributed (Garcia-Longoria et al., 2015; Hellgren et al., 2015; Santiago-Alarcon et al., 2012; Valkiūnas, 2005), many other haemosporidian parasites have a restricted distribution due to host specificity and geographical barriers (Clark and Clegg, 2017; Ellis et al., 2018; Gupta et al., 2019; Hellgren et al., 2009). Compared to resident bird species, migratory species are exposed to a larger array of parasites and vectors as they cross-over through different habitats and environments (Inumaru et al., 2017; Pulgarín-R et al., 2019; Ramey et al., 2015; Soares et al., 2020; Waldenström et al., 2002). The vector and parasite fauna that migratory birds come in contact with will vary depending on their migration route and strategy. As mentioned above (see section 4.2), Swinhoe's snipes has wider distribution compared to Latham's snipes (Frith et al., 1977; Hayman et al., 1986; Leader and Carey, 2003; Morozov, 2004; Ura, 2007), so they may encounter different parasites and may collectively exhibit a greater diversity in parasite lineages. Furthermore, the Swinhoe's snipes captured in this study probably consist of various populations that inhabit different areas, as the differences in lineage composition of Swinhoe's snipes between individuals captured in central Japan and southwest Japan suggests (Table 3). Along with the difference in prevalence between these two areas, this difference in lineage composition may also be a result of differing migratory routes.

### 4.5 Timing and area of transmission

Six of the seven lineages were detected from juveniles. These individuals were born in the preceding breeding season and were on their first migration towards their wintering grounds. This means that they have never experienced the wintering grounds and hence, the detected parasites were transmitted between their breeding grounds and their captured locations during migration.

Furthermore, *Plasmodium circumflexum* pSW5, which has been found in various parts of the world (Bensch et al., 2007; Biedrzycka et al., 2015; Inumaru et al., 2017; Ramey et al., 2016; Tanigawa et al., 2013; Waldenström et al., 2002), was detected in this study and its only known vectors are *Culiseta* spp. mosquitoes (Meyer and Bennett, 1976; Smith et al., 2019; Valkiūnas, 2005), which inhabit the Holarctic (Medvedev, 2009). However, within Japan, this mosquito genus can only be found in the northernmost areas (Ejiri et al., 2011; Maekawa et al., 2016; Ono, 1969). It is therefore thought that pSW5 cannot be transmitted in most of Japan and can only be transmitted in the northernmost areas inhabited by *Culiseta* spp. mosquitoes (Inumaru et al., 2017). Hence, this *Plasmodium* species was most likely transmitted to snipes in northernmost Japan or continental areas from Russia to Mongolia.

### 4.6 Morphological effects to host birds

In addition to lethal effects (Bennett et al., 1993; Lee et al., 2018; Valkiūnas, 2005; Van Riper et al., 1986; Vanstreels et al., 2016), haemosporidian parasites can provoke other negative effects on their avian hosts (Coon et al., 2016; Dunn et al., 2013; Fleskes et al., 2017; Höglund et al., 2017; Marzal et al, 2008, 2013; Merino et al., 2000; Morales et al., 2007; Palinauskas et al., 2008). Contrary to expectations, there was no difference among infected and uninfected individuals among all measurements for both Swinhoe's snipes and Latham's snipes of both sexes. Some studies demonstrating negative effects of infection on body condition has suggested that these effects may be linked with not only infection, but also with other conditions such as immune response, molt and availability of resources (Cornet et al., 2014; Dunn et al., 2013; Hatchwell et al., 2001; Morales et al., 2007; Palinauskas et al., 2020). In addition, infections at low intensities may show little to no effects on the host bird (Hahn et al., 2018; Palinauskas et al., 2020). Hence, the parasite intensity might have potentially been light enough that no observable differences in morphological traits were observed. Another possibility is that heavily infected individuals may have not been sampled, as these individuals are less likely to survive the physiological stress of long-distance migration (i.e. migratory culling) (Altizer et al., 2011; Dawson and Bortolotti, 2000; Marzal et al., 2016; Satterfield et al., 2018). Consequently, only snipes with light parasite intensities that were able to cope with the infection during migration might have been sampled. Parasite intensity was not confirmed in this study and will need to be confirmed in future studies.

## 5 Conclusion

While there are conservation projects for the Latham's snipe, information on the biology of these snipes including migration is still insufficient. We investigated the prevalence and genetic diversity of avian haemosporidia in three *Gallinago* snipe species of Japan for the first time in effort to expand knowledge on these species and the parasites they carry. Although reasons could not be completely understood, various possibilities to explain varying parasite prevalence were discussed including habitat, migratory and physiological differences. While some lineages suggested possible locations of transmission, other lineages were newly detected and require further studies to unveil the distribution and transmission area. We found no relation between parasite infection and morphological features of birds. However, in order to further contribute to conservational actions, more work is necessary addressing a better image of the area of transmission and possible physiological effects including virulence. Linking studies between biological research, such as on the habitat and migratory routes of these snipes, and parasitology research are anticipated.

## Declaration of competing interest

The authors declare that they have no conflict of interest.
